# Habitat disturbance results in chronic stress and impaired health status in forest-dwelling paleotropical bats

**DOI:** 10.1093/conphys/cox020

**Published:** 2017-04-05

**Authors:** Anne Seltmann, Gábor Á. Czirják, Alexandre Courtiol, Henry Bernard, Matthew J. Struebig, Christian C. Voigt

**Affiliations:** 1 Department of Evolutionary Ecology, Leibniz Institute for Zoo and Wildlife Research, Alfred-Kowalke-Str. 17, 10315 Berlin, Germany; 2 Institute of Biology, Freie Universität Berlin, Takustr. 3, 14195 Berlin, Germany; 3 Department of Wildlife Diseases, Leibniz Institute for Zoo and Wildlife Research, Alfred-Kowalke-Str. 17, 10315 Berlin, Germany; 4 Department of Evolutionary Genetics, Leibniz Institute for Zoo and Wildlife Research, Alfred-Kowalke-Str. 17, 10315 Berlin, Germany; 5 Institute for Tropical Biology and Conservation, Universiti Malaysia Sabah, Jalan UMS, 88400 Kota Kinabalu, Sabah, Malaysia; 6 Durrell Institute of Conservation and Ecology (DICE), School of Anthropology and Conservation, University of Kent, Canterbury, Kent CT2 7NR, UK

**Keywords:** Anthropogenic disturbance, bats, body mass, chronic stress, fragmentation, white blood cell count

## Abstract

Anthropogenic habitat disturbance is a major threat to biodiversity worldwide. Yet, before population declines are detectable, individuals may suffer from chronic stress and impaired immunity in disturbed habitats, making them more susceptible to pathogens and adverse weather conditions. Here, we tested in a paleotropical forest with ongoing logging and fragmentation, whether habitat disturbance influences the body mass and immunity of bats. We measured and compared body mass, chronic stress (indicated by neutrophil to lymphocyte ratios) and the number of circulating immune cells between several bat species with different roost types living in recovering areas, actively logged forests, and fragmented forests in Sabah, Malaysia. In a cave-roosting species, chronic stress levels were higher in individuals from fragmented habitats compared with conspecifics from actively logged areas. Foliage-roosting species showed a reduced body mass and decrease in total white blood cell counts in actively logged areas and fragmented forests compared with conspecifics living in recovering habitats. Our study highlights that habitat disturbance may have species-specific effects on chronic stress and immunity in bats that are potentially related to the roost type. We identified foliage-roosting species as particularly sensitive to forest habitat deterioration. These species may face a heightened extinction risk in the near future if anthropogenic habitat alterations continue.

## Teaser

Encroachment of humans into pristine habitats is a major threat to biodiversity. We found that some paleotropical bat species from disturbed forests suffered from chronic stress and an impaired immune system, suggesting that allostatic overload may play a pivotal role in the extinction of wildlife species.

## Introduction

Habitat degradation, loss and fragmentation are widespread causes of biodiversity loss worldwide (Barnosky *et al*., 2011; Gibson *et al*., 2011; Laurance *et al*., 2014). Considering the pace at which habitats are altered by humans, it is essential to understand how anthropogenic land-use changes affect wildlife species and whether this impact could as well influence human and animal health (Ellis *et al*., 2012). Encroachment of humans into formerly pristine habitats often leads to reduced wildlife populations and local extinction of species (Newbold *et al*., 2014; Haddad *et al*., 2015; Meyer *et al*., 2016). However, human disturbances also affect wildlife at the individual level (Ellis *et al*., 2012). Focusing on individual physiology may help us to estimate the consequences of environmental stressors on wildlife before the effects begin to manifest in decreased population sizes (Davis *et al*., 2008).

Indeed, previous studies have observed chronic stress and an impaired body condition in various wildlife species from disturbed habitats, suggesting that anthropogenic perturbations may become a major stressor for wildlife species (reviewed by Romero, 2004; Ellis *et al*., 2012). This is because habitat fragmentation may force individuals of mobile taxa to use more than one patch for foraging. By using multiple patches, individuals are thus exposed to a higher predation risk and have to spend more energy and time to meet their food requirement (Hinsley, 2000). Additionally, the quality of the habitat may decline, exacerbating the limitation of food resources (Ellis *et al*., 2012). Further, habitat deterioration and fragmentation may result in the loss of shelter, and also an increased exposure to abiotic factors such as wind and rain, especially at habitat edges (Ellis *et al*., 2012).

Although chronic stress caused by natural stressors (e.g. predation pressure) can be adaptive in wildlife populations (Boonstra, 2013), it also has the potential to lead to an impaired body condition and to permanently disrupt the homeostasis of animals (Romero, 2002, 2004; Wingfield, 2005; Wikelski and Cooke, 2006; Romero and Wikelski, 2010; Dickens and Romero, 2013). For example, chronically elevated plasma levels of circulating glucocorticoid hormones may suppress the immune system of animals, decrease their survival, their growth and reproductive function (Dhabhar *et al*., 1996; Wingfield *et al*., 1997; Buchanan, 2000; Sapolsky *et al*., 2000). Thus, animals that are not resilient to anthropogenic perturbations may suffer from chronic stress and, as a consequence, from impaired immunity (Suorsa *et al*., 2004). For example, Allen *et al*. (2009) found that bats roosting at human-made bridges experienced physiological stress, which resulted in reduced immune system functioning. An impaired immune system may then result in increased susceptibility, shedding of pathogens and decreased survival rates (Demas *et al*., 2011).

Bats (Chiroptera) are a promising taxon to study the effects of forest degradation, habitat loss and fragmentation on body condition, chronic stress and immunity, since bats are known to be highly sensitive to the effects of environmental change (Jones *et al*., 2009; Struebig *et al*., 2011; Meyer, *et al*., 2016). Furthermore, more than 100 different viruses have been associated with Chiroptera, including several particularly virulent ones, such as rabies, Severe Acute Respiratory Syndrome (SARS)-like coronavirus, Nipah, Hendra, Ebola and Marburg (Chua *et al*., 2000; Leroy *et al*., 2005; Li *et al*., 2005; Towner *et al*., 2009; Wibbelt *et al*., 2010; Wang *et al*., 2011; Baker *et al*., 2013; Brook and Dobson, 2015). Bats harbor on average the highest number of viruses than any other taxa per species, probably due to their extraordinary longevity and high levels of interspecific viral transmission (Luis *et al*., 2013). Surprisingly, bats often do not show clinical signs of viral infections (Wibbelt *et al*., 2010; Epstein and Field, 2016). Despite their potential role as viral reservoirs, there is almost no information on the immune function of bat species, and an understanding of the ecological and social factors influencing the intra- and interspecific immune differences between taxa is lacking (but see Allen *et al*., 2009; Schneeberger *et al*., 2013a).

In our study, we assessed the impact of anthropogenic disturbances on body mass in eight, and on immunological parameters in three forest bat species in Malaysian Borneo, each of which utilize the cluttered forest environment for foraging. In contrast to bat species utilizing forest edges, these forest specialists might suffer from fragmentation in particular because of decreased availability of potential prey insects and roost sites in the forest interior. Other studies in the Paleo- and Neotropics have already shown that feeding activity and abundance of some forest bat species is lower in smaller compared with larger fragments or continuous forests (Meyer *et al*., 2008; Estrada-Villegas *et al*., 2010; Struebig *et al*., 2011). Therefore, we predicted that foliage-roosting bats from actively logged areas and fragmented forest exhibit lower body masses, signs of chronic stress, assessed by the neutrophils to lymphocytes (N/L)-ratio (Davis *et al*., 2008), and impaired immunity, measured as total white blood cell counts, compared with bats of recovering forest. In cave-roosting bats, we predicted smaller effects due to their substantial larger home range sizes and more permanent roosting sites compared with foliage-roosting species, which may enable cave-roosting bats to cover wider, disturbed and intact areas by flying longer distances from respective roosts to their foraging site (Struebig *et al*., 2013).

## Methods

### Study site and species

**Figure 1:**
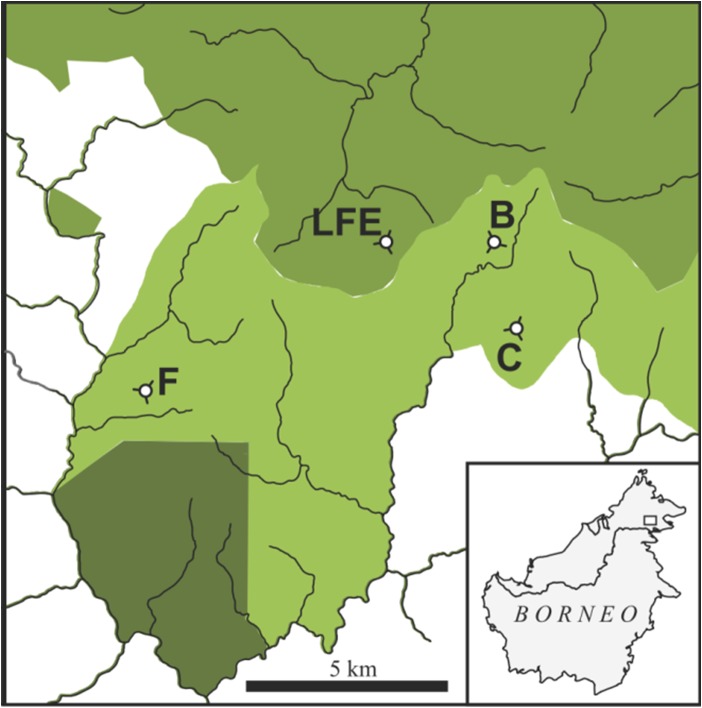
Location of forest sites sampled for bats at the Stability of Altered Forest Ecosystems Project in Sabah, Borneo. Prior to sampling all sites had been logged multiple times, with sites B, C and F (light shading) experiencing much higher extraction rates than site LFE (intermediate shading), and old growth forest in the southwest (dark shading). Sites were sampled for bats multiple times during the final round of logging and subsequent fragmentation of sites B, C and F. Site LFE remained as a control site throughout the study with no further logging or fragmentation activity at or around this locality. The oil palm plantation matrix is shown in white and lines indicate rivers.

The study was conducted within the SAFE project (Stability of Altered Forest Ecosystems, www.safeproject.net), a 7200 ha landscape fragmentation experiment established in Sabah, Borneo. The SAFE landscape comprises logged over dipterocarp rainforest, some of which is being converted to oil palm plantation, leaving behind a network of disturbed forest fragments. Thus, the landscape represents a land-use transition common across much of Southeast Asia (Fitzherbert *et al*., 2008; Gaveau *et al*., 2014; Marlier *et al*., 2015). All sample locations were situated within 10 km of a research camp at N4.73 E117.60 (Fig. 1). Much of the landscape had been logged twice prior to our study and the coupes allocated for conversion to oil palm had been heavily logged multiple times before (Struebig *et al*., 2013). At the time of sampling, these areas were experiencing a final harvest prior to conversion and were at the early stages of fragmentation, with large areas devoid of any tree cover, ultimately resulting in ten isolated plots (e.g. B, C, F and LFE). We sampled bats multiple times at various sites across this disturbance gradient during the conversion process: in plot B, C, F and LFE. These plots were located 2–10 km apart from each other, exceeding the mean home range size of foliage-roosting insectivorous bat species (Struebig *et al*., 2013). LFE is a twice-logged site at which logging operations ceased in the late 1990s leaving time for substantial recovery of a tall dipterocarp forest; and sites B, C and F had experienced additionally logging prior to the onset of the study, and then experienced heavy logging and fragmentation throughout the 2-year study period. We categorized these sites into three disturbance levels according to human logging activity at the time of sampling: (1) Relatively undisturbed (only presence of SAFE-affiliated researchers with minimal impact), hereafter ‘Recovering forest’: LFE, B_1_ and C_1_, whereby subscript denotes the order of sampling. (2) Sites that were heavily logged at the time of sampling were categorized as ‘Actively logged forest’: B_2_, C_2_ and F_1_. After logging was completed and thus sites relatively undisturbed, site F (i.e. F_2_) was categorized as ‘Fragmented’ (for sites B and C logging was still underway at the end of sampling). Data collection took place between July and September in 2014 and 2015, with additional sampling period between March and April in 2015 (Table 1).

**Table 1:** Study sites, habitat type and sampling year

	Recovering forest	Actively logged forest	Fragmented forest
LFE	2014, 2015	–	–
B	2014, 2015	2015	–
C	2014, 2015	2015	–
F	–	2014, 2015	2015

The landscape has a well characterized insectivorous bat fauna, which is known to have experienced a substantial shift in assemblage composition in response to past logging (Struebig *et al*., 2013). We recorded body measurements of all adult individuals captured, but selected study species from the families Vespertilionidae (subfamily: Kerivoulinae), Hipposideridae and Rhinolophidae, which were sufficiently abundant across the landscape to warrant sufficient sample size. Within the subfamily Kerivoulinae (woolly bats), we studied the following congeneric bats: *Kervivoula intermedia*, *K. papillosa* and *K. hardwickii*. Within the family Hipposideridae (leaf-nosed bats), we focused on the congeneric species *Hipposideros cervinus* and *H. dyacorum*, and within the family of Rhinolophidae (horseshoe bats) on *Rhinolophus sedulus*, *R. trifoliatus* and *R. borneensis.* All species of the subfamily Kerivoulinae are thought to roost solitarily in foliage, except for *K. papillosa* which roosts in tree cavities living in dynamic fission-fusion societies (Payne *et al*., 1985; Kingston *et al*., 2006). *Hipposideros cervinus, H. dyacorum* and *R. borneensis* form colonies roosting in caves or cave-like structures, whereas *R. sedulus* and *R. trifoliatus* are solitary or monogamous, roosting in trees (Payne *et al*., 1985; Kingston *et al*., 2006). All species are small, insectivorous bats with body masses ranging between 3 and 16 g (Payne *et al*., 1985). In 2011/2012, prior to our sampling, bat abundance for our study species was moderate to high in all sites, but richness was lower in the repeatedly logged sites (B, C, F) compared to our recovering twice logged site (LFE, Table 2, Struebig *et al*., 2013).

**Table 2:** Bat capture rates (individuals/harp trap night) at sampling sites prior to the study in 2011/2012 (Struebig *et al*., 2013) and this study period (2014/2015)

	Prior to conversion (2011/2012)				Recovering forest (2014/2015)			Actively logged forest (2014/2015)			Fragmented forest (2015)
Species/plot	B	C	F	LFE	B	C	LFE	B	C	F	F
*Hipposideros cervinus*	0.74	0.88	0.86	0.45	0.22	0.68	0.23	0.19	0.47	0.1	0.2
*Hipposideros dyacorum*	0	0.02	0.76	0.02	0	0.08	0.04	0.02	0	0.25	0.43
*Kerivoula hardwickii*	0.40	0.46	0.26	0.31	0.5	0.42	0.35	0.12	0.11	0.15	0.17
*Kerivoula intermedia*	0.71	0.29	0.07	0.26	0.77	0.45	0.54	0.31	0.19	0.3	0.36
*Kerivoula papillosa*	0.17	0	0.12	0.07	0.02	0	0.07	0.02	0	0.03	0.04
*Rhinolophus borneensis*	0.12	0.24	0.17	0.10	0.02	0.1	0.04	0.05	0.06	0.05	0.16
*Rhinolophus sedulus*	0.33	0.17	0.05	0.29	0.04	0.05	0.11	0.05	0.06	0.03	0
*Rhinolophus trifoliatus*	0.29	0.43	0.33	0.14	0.25	0.25	0.09	0.19	0.06	0.25	0.25

### Bat capture

In the morning hours after sunrise, we set up six harp traps (Museum Zoologicum Bogoriense, Bogor, Indonesia) along established trails in the forest, with a minimum distance of 30–100 m. Harp traps are an established sampling method for capturing forest bats and are particularly effective in the paleotropics (Kingston *et al*., 2003; Kunz *et al*., 2009). Between subsequent nights, we moved traps to new positions, resulting in a total of 15–20 positions per site and survey. The total sampling effort over the study period was 321 harp trap nights.

We checked traps at 1900 and at 0700 the following day. Bats were retrieved from harp traps and transported back to the camp in individual cloth bags for processing, with the exception of fruit bats (Pteropodidae), individuals of *H. cervinus* (known to be highly sensitive towards handling), juveniles, pregnant or lactating females of all species. These individuals were instead processed on site and released as soon as possible at the point of capture, typically within 2 h. Pregnant females were excluded from analyses of body mass because the fetus influences body mass to an unknown extent. We identified species according to Kingston *et al*. (2006) and Struebig and Sujarno (2006). Juveniles were distinguished from adults by the epiphyseal closure of phalanges (Kunz and Anthony, 1982). We classified the reproductive status of females (non-reproductive, pregnant, lactating or post-lactating) by abdominal palpation and visual inspection of the teats and surrounding area.

We recorded body mass (g) by using a spring balance (Pesola balance, Switzerland, accuracy = 0.25 g) and length of forearm (mm) using a caliper (Wiha Werkzeuge GmbH, Schonach, Germany). Further, we noted if the bat had consumed a large visible amount of prey based on visual inspection of the torso. We marked all adult bats with a uniquely coded forearm band of 2.9 or 4.2 mm, depending on size of the bat (Porzana Limited, East Sussex, UK) as described in Kunz and Weise (2009). Blood samples were collected only from the rhinolophid species as these taxa were larger than Kerivoulinae and Hipposideridae, and thus sufficient blood could be collected without undue harm to the individual. From rhinolophid species we collected a small quantity of whole blood (max. 140 µl, <1 % of the body mass) in a heparinized capillary (Paul Marienfeld GmbH & Co.KG, Lauda-Koenigshofen, Germany) by puncturing the antebrachial vein as described in Voigt and Cruz-Neto (2009). For venipuncture we used sterile needles (Henry Schein Inc., Melville, New York, USA). The blood sample was transferred with a micropipetter (Hirschmann Laborgeräte GmbH & Co.KG, Eberstadt, Germany) to a tube, and a subsample of 3 µl was used to produce a blood smear on glass slides (Carl Roth GmbH & Co.KG, Karlsruhe, Germany). All bats were released at the capture site within 12 h. Although we took measurements for all adult bats captured, only data of the eight focus species are used in analyses as justified above.

### Ethics statement

Our study and export of samples was authorized by the scientific committee of the Sabah Biodiversity Center, Sabah, Malaysia (JKM/MBS.1000-2/2 (317); JKM/MBS.1000-2/3 JLD.2 (16); JKM/MBS.1000-2/2 JLD.3 (153)), as well as the SAFE project and complies with the laws of Malaysia and Germany.

### White blood cell counts

We stained blood smears with May-Gruenwald's solution (#T863.2, Carl Roth GmbH & Co.KG, Karlsruhe, Germany) and Giemsa (#T862.1, Carl Roth GmbH & Co.KG, Karlsruhe, Germany). Blood smears were analyzed with a microscope under oil immersion at a 100× magnification. Some samples were suboptimal due to adverse weather conditions (e.g. high humidity causing mold) and had to be discarded without analyses. Blood smears were analyzed blindly with respect to the identity of individuals and conducted by the same person (A.S.).

Total white blood cell (TWBC) counts were estimated manually by the mean number of leukocytes per visual field using the total count of leukocytes in 10 fields, or the number of fields that was necessary to examine until a single leukocyte was observed (Schneeberger *et al*., 2013b). For the differential white blood cell (DWBC) counts, we counted 100 (or 50 in few cases) leukocytes and identified the different types of leukocytes by size, color, shape and cytoplasmic contents. From this, we calculated relative numbers (%) of lymphocytes, neutrophils, eosinophils, monocytes and basophils and also the N/L-ratio, which was used as an estimate for chronic stress (Davis *et al*., 2008).

### Statistical analyses

We performed all statistical analyses using the statistical software R version 3.3.1 (R Core Team, 2016). We conducted two-tailed tests (when applicable) and set the level of significance to *α* = 0.05. Means and standard errors are given in the format X ± SE, unless stated otherwise.

Due to the large sample size of the dataset for body mass (*N* = 443), we studied the influence of different predictor variables on the body mass using a parametric method—the linear model. We included the following predictor variables in the linear model: the interaction between species (8 levels) and habitat type (3 levels: recovering forest, actively logged forest or fragmented forest), the standardized deviation from the species-specific mean forearm length (continuous: *z*-score) to control for intraspecific variation in body size, plot identity (4 levels: B, C, F and LFE), reproductive status and sex (3 levels: male, lactating and non-reproducing females), recent feeding status (2 levels: fed, non-fed), season (2 levels: dry season: March-April, characterized by a mean monthly precipitation of 77 mm; beginning of rainy season: July–September, characterized by a mean monthly precipitation of 170 mm, personal communication from Prof. R. Walsh, Swansea University, UK) and year (2014, 2015). The body mass and the forearm length (as *z*-scores) were log-transformed before the analysis. We did not consider the interaction between species and the forearm length because within our double logarithmic approach the regression coefficient associated to the forearm length corresponds to an allometric coefficient that has been shown to present little variation between bat species (Norberg, 1981). The total number of parameters considered in the linear model was thus 31. As required, the residuals of our model were homoscedastic and normally distributed. We used the function *lm* readily available in R to fit our linear model, followed by *Anova* from the package ‘car’ (Fox and Weisberg, 2011) to assess the significance of predictor variables using marginal (type II) *F*-tests. If a non-continuous predictor had a significant effect on the response variable, we performed post hoc analyses using the function *glht* from the package ‘multcomp’ (Hothorn *et al*., 2008), which performs generalized linear hypotheses testing (GLHT). Because we were interested in the effect of roost type on body mass in different habitats, we grouped the species according to their roosting habit in cave- (*H. cervinus*, *H. dyacorum*, *R. borneensis*) and foliage-roosting species (*K. hardwickii*, *K. intermedia*, *K. papillosa*, *R. trifoliatus*, *R. sedulus*) prior to performing post hoc analyses.

Our sample size was sufficient to fulfill the recommended minimal amount of data points for our model to avoid overfitting (i.e. 10 × *k*; e.g. Harrel, 2001 or 50 + 8 × *k*; Field, 2005 with *k* = 31). Our linear model should also not suffer from potential problems arising from multi-collinearity (i.e. correlation between the predictors). We tested this possibility by computing generalized variance inflation factors (specifically, the square of the generalized variance inflation factors scaled for the number of degree of freedom, or [GVIF^(1/(2*Df))]^2) according to Fox and Monette (1992). The maximum value obtained was 3.5 which is below the critical threshold of 4–10 usually mentioned in the literature (reviewed by O'Brien, 2007).

As some pairs of bat species are more related to each other than others in our sample, we also fitted the same linear model as a phylogenetic generalized least squares (PGLS) using the R packages ‘ape’, ‘geiger’ and ‘phytools’ (Paradis *et al*., 2004; Harmon *et al*., 2008; Revell, 2012) in order to test for the presence of a phylogenetic signal. We considered the Brownian, the Grafen and the Pagel's lambda correlations structure in the PGLS (Felsenstein, 1985; Grafen, 1989; Martins and Hansen, 1997), and based our phylogenetic information on Khan *et al*. (2010) and Sazali *et al*. (2011). Because we did not find evidence for a phylogenetic signal, we only present the linear model in this paper for the reason of simplicity.

Due to small sample sizes, we analyzed differences in TWBC counts and in N/L-ratio between habitat types within rhinolophid species using the non-parametric Kruskal–Wallis-test followed by the Dunn's test for post hoc analyses (R package ‘dunn.test’; Dinno, 2015). We used the Bonferroni method to perform multiple-comparison adjustments during the Dunn's test.

## Results

In total, we obtained data from 443 adult individuals of the eight target species (see Table 2). Data on body mass were available for all 443 individuals (see Table 3). For rhinolophid species, we obtained differential and TWBC counts for 85 and 82 individual bats, respectively.

**Table 3:** Body masses and roosting ecology of study species in each habitat type (values indicate mean **±**SE (g) and sample size in parentheses)

Species	Roosting ecology	Recovering forest	Actively logged forest	Fragmented forest
*Rhinolophus trifoliatus*	Foliage	13.8 ± 1.6 (26)	14.0 ± 1.7 (20)	13.2 ± 1.5 (10)
*Hipposideros cervinus*	Cave	9.8 ± 1.4 (52)	10.4 ± 1.1 (29)	10.3 ± 1.0 (10)
*Rhinolophus sedulus*	Foliage	10.9 ± 2.6 (9)	9.4 ± 0.9 (5)	NA
*Kerivoula papillosa*	Foliage	9.5 ± 1.5 (6)	11.5 ± 2.1 (2)	7.5 ± 0.7 (2)
*Rhinolophus borneensis*	Cave	8.8 ± 1.1 (7)	9.3 ± 0.7 (7)	9.1 ± 1.1 (7)
*Hipposideros dyacorum*	Cave	7 ± 0.4 (5)	7.3 ± 1.4 (11)	6.9 ± 0.9 (21)
*Kerivoula hardwickii*	Foliage	3.7 ± 0.4 (63)	3.7 ± 0.4 (16)	3.2 ± 0.2 (9)
*Kerivoula intermedia*	Foliage	3.4 ± 0.4 (78)	3.3 ± 0.4 (32)	3.0 ± 0.3 (19)

Species are ordered by size (largest to smallest).

### Body mass in relation to habitat type

**Figure 2:**
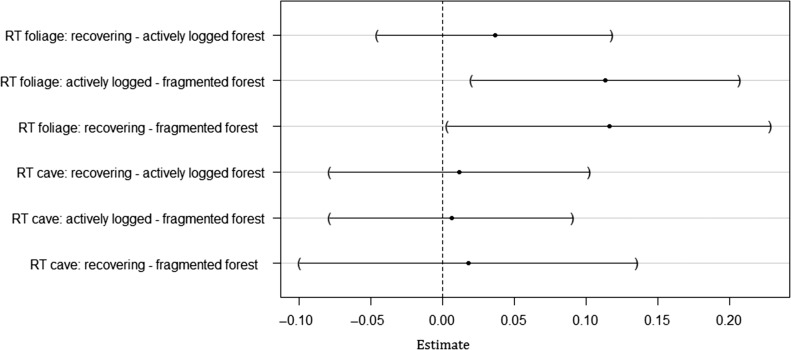
Post hoc pairwise comparisons between predicted body mass (log) for different habitat types within roost type (RT). The figure shows the difference in estimate (dot) and the 95% confidence interval of the difference (line) for each pairwise comparison.

Overall, the linear model for body mass (log) accounted for 97% of the total variation in body mass. Body mass varied significantly depending on the habitat-species combination (Anova, *F* = 1.86, df_1_ = 13, df_2_ = 401, *P* = 0.033). In cave-roosting bats, there were no significant differences in body mass across habitat types (GLHT, absolute *t*-values < 0.40, *P* > 0.99 for all pairwise comparison between habitat types, Fig. 2). In foliage-roosting species, body masses of individual bats were significantly lower in fragmented compared to actively logged forests (GLHT, exp (Estimate) = −1.12 g, **t**-value = −3.14, *P* = 0.01) and higher in recovering than in fragmented forest (GLHT, exp (Estimate) = 1.12 g, **t**-value = 2.67, *P* = 0.04). Foliage-roosting bats captured in recovering forest were slightly heavier than those captured in actively logged forest, although the difference was not significant (GLHT, exp (Estimate) = 1.04 g, **t**-value = 1.15, *P* = 0.70).

As expected, body mass significantly increased with the *z*-score of forearm length (log) (Anova, *F* = 34.35, df_1_ = 1, df_2_ = 401, *P* < 0.001). The location of capture (plot) had a significant effect on body mass (Anova, *F* = 4.32, df_1_ = 3, df_2_ = 401, *P* = 0.005): In plot B, bats weighed significantly more than in plot LFE (GLHT, Estimate = 0.06, *t*-value = 3.57, *P* = 0.002). There were no significant differences in body mass between the other plots (GLHT, all *t*-values < 2.3, all *P* > 0.1). Bats that had clearly eaten recently were significantly heavier than bats that did not recently feed before capture (Anova, *F* = 28.04, df_1_ = 1, df_2_ = 401, *P* < 0.001). Body mass varied with reproductive condition (Anova, *F* = 5.43, df_1_ = 2, df_2_ = 401, *P* = 0.005): Non-reproducing females weighed significantly less than males (GLHT, Estimate = −0.04, *t*-value = 3.27, *P* = 0.003). There were no significant differences in body mass between lactating and non-reproducing females and males, respectively (GLHT, all *t*-values <0.04, all *P* > 0.36). In addition, body mass was significantly higher in bats captured during the beginning of the rainy season compared with those being captured during the dry season (Anova, *F* = 14.42, df_1_ = 1, df_2_ = 401, *P* < 0.001). We did not detect any annual fluctuations in body mass (Anova, *F* = < 0.01, df_1_ = 1, df_2_ = 401, *P* = 0.99).

### Stress and immunological measures in relation to forest categories

**Figure 3:**
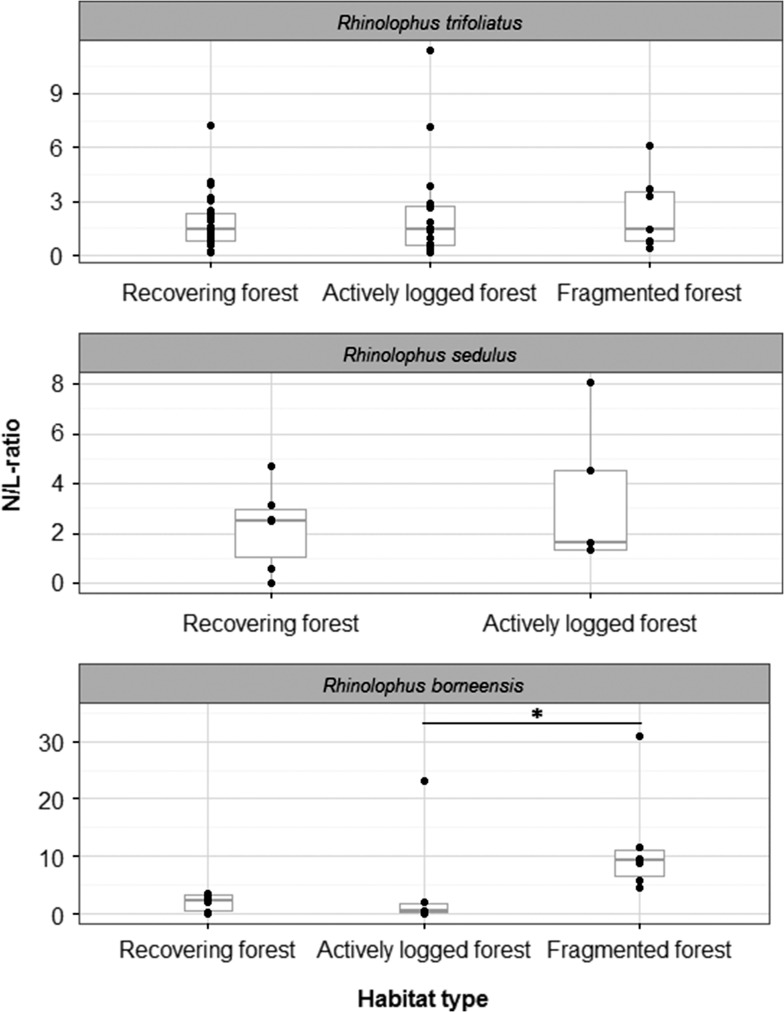
Association between N/L-ratio and habitat type for *Rhinolophus borneensis*,*R. sedulus* and* R. trifoliatus*. The figure shows data points (dots), medians (bold line) and 25–75% percentiles (box). The N/L-ratio does not significantly differ between habitat types in *R. sedulus* (*n* = 11) and *R. trifoliatus* (*n* = 55). In *R. borneensis* (*n*= 19), individuals living in fragmented forest had significantly higher N/L-ratios than those in actively logged forest. For reasons of clearer representation one data point in the dataset of *R. borneensis* (N/L-ratio = 47.5, habitat type: recovering forest) is not depicted.

We measured the N/L-ratio (a proxy for chronic stress levels) in three congeneric species. *Rhinolophus trifoliatus* and *R. sedulus* had N/L-ratios with relatively low variation of 0.18–11.38 (median 1.48) and 0–8.09 (median 2.48), respectively, whereas *R. borneensis* showed higher variation in N/L-ratio (0.06–47.5, median 3.36). The overall results of the DWBC counts can be found in Table 4. Kruskal–Wallis (KW)-tests within each species revealed that there was no significant difference in the N/L-ratio between habitat types in *R. trifoliatus* and *R. sedulus* (*R. trifoliatus*: KW chi-squared = 0.19, df = 2, *P* = 0.91, *n* = 55; *R. sedulus*: KW chi-squared = 0.13, df = 1, *P* = 0.72, *n* = 11; Fig. 3). In *R. borneensis*, we found that individuals living in fragmented forest had significantly higher N/L-ratios than conspecifics from actively logged areas (fragmented forest: 11.91 ± 9.69, active logging areas: 5.25 ± 9.24, recovering forest: 8.51 ± 17.25; KW-tests, KW chi-squared = 6.52, df = 2, *P* = 0.04, *n* = 19; Dunn's test between fragmented and actively logged forest, *Z* = −2.46, *P* = 0.02, Dunn's test between recovering and fragmented forest, *Z* = −1.86, *P* = 0.1, Dunn's test between recovering and actively logged forest, *Z* = 0.7, *P* = 0.72).

**Table 4:** Differential white blood cell counts in *Rhinolophus trifoliatus*, *R. sedulus* and *R. borneensis*. Values indicate mean ± SE (%)

Species (sample size)	Neutrophils	Lymphocytes	Eosinophils	Monocytes	Basophils
*Rhinolophus trifoliatus* (*n* = 55)	54.42 ± 19.82	41.75 ± 20.05	0.42 ± 1.07	2.38 ± 2.31	0.07 ± 0.26
*Rhinolophus sedulus* (*n* = 11)	61.09 ± 25.31	36.73 ± 24.28	0.54 ± 1.04	1.55 ± 1.63	0.09 ± 0.30
*Rhinolophus borneensis* (*n* = 19)	62.05 ± 31.97	35.13 ± 31.90	1.21 ± 1.51	1.39 ± 1.48	0.21 ± 0.54

**Figure 4:**
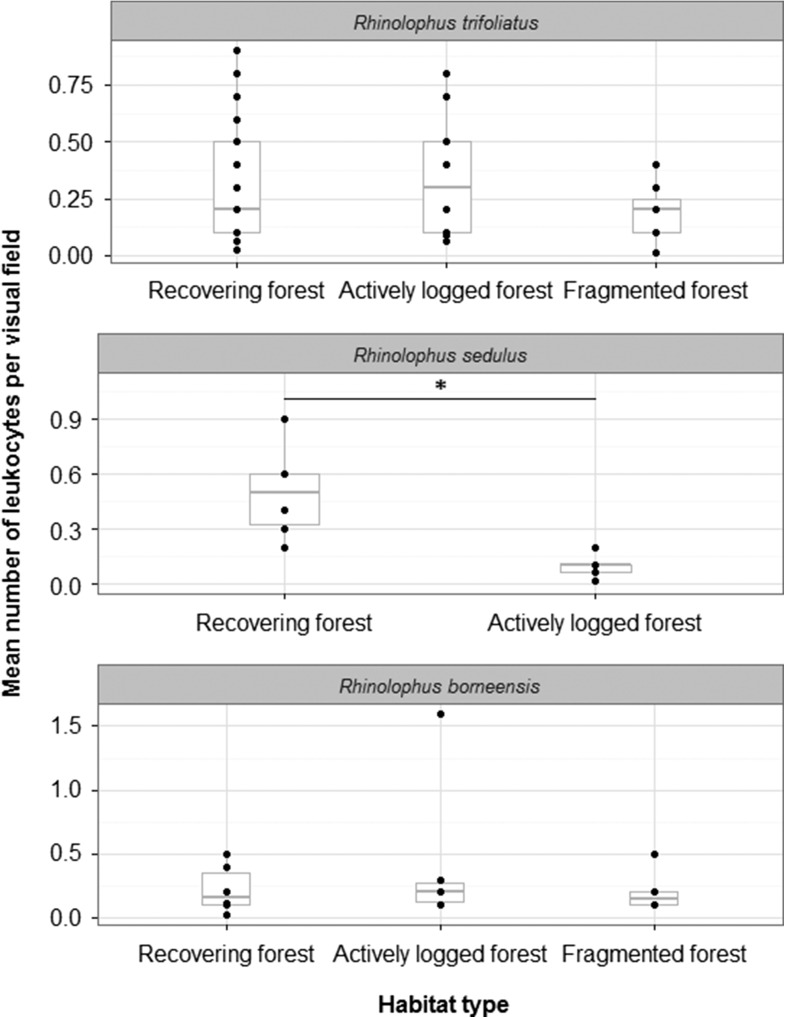
Association between mean number of leukocytes per optical field and habitat type for *Rhinolophus borneensis*, *R. sedulus* and *R. trifoliatus.* The figure shows data points (dots), medians (bold line) and 25–75% percentiles (box). There is no significant difference in the number of leukocytes between habitat types in *Rhinolophus trifoliatus* (*n* = 53) and *R. borneensis* (*n* = 18), while numbers of leukocytes are significantly lower in individuals from actively logged than from recovering forest in *R. sedulus* (*n* = 11).

The mean number of leukocytes per optical field was similar for all study species (*R. trifoliatus*: 0.31 ± 0.2, *R. sedulus*: 0.32 ± 0.28, *R. borneensis*: 0.28 ± 0.36). Leukocyte number was not related to habitat type in *R. trifoliatus* and *R. borneensis* (KW-tests, *R. trifoliatus*: KW chi-squared = 1.74, df = 2, *P* = 0.42, *n* = 53; *R. borneensis*: KW chi-squared = 0.38, df = 2, *P* = 0.83, *n* = 18; Fig. 4). We found significantly lower numbers of leukocytes in *R. sedulus* captured in logging areas compared with conspecifics from recovering forests (actively logged forest: 0.1 ± 0.07, recovering forest: 0.5 ± 0.25; Mann–Whitney *U* test, *W* = 29.5, *P* = 0.01, *n* = 11). *Rhinolophus sedulus* was absent from fragmented forest.

## Discussion

This study aimed to evaluate the impact of habitat disturbance on body mass, chronic stress and cellular immunity in forest-dwelling paleotropical bats. Forest disturbance was associated with a reduced individual body mass in foliage-roosting species. In one cave-roosting species (*Rhinolophus borneensis*), we found elevated N/L-ratios in individuals from fragmented forest compared to individuals from active logging areas, indicating chronic stress. In a foliage-roosting species (*R. sedulus*), we found reduced white blood cell numbers in actively logged compared to less disturbed recovering forest. Keeping in mind that the biological differences might be small, we may not have been able to detect significant differences due to low sample sizes in some of the study species.

### Body mass and habitat alteration

Anthropogenic habitat disturbance had a roost type-specific effect on bat body mass in our study. In foliage-roosting species, body mass of individual bats decreased with increasing disturbance level. In cave-roosting species, the body mass was not significantly associated with habitat type, although trends were similar to foliage-roosting species. Cave-roosting species may benefit from increased edge habitats and decreased abundance of individuals of foliage-roosting species, resulting in reduced competition for food due to their relatively large home range size associated with central-place foraging (Struebig *et al*., 2013). Confirming our findings, Struebig *et al*. (2013) found that some cave-roosting species such as *H. cervinus* were highly abundant across heavily disturbed sites.

The observation that habitat alteration affects body mass supports the findings of other studies, although variation exists across species. Most work in this direction has so far been conducted in birds. For example, in montane white-eyes (*Zosterops poliogaster*) body mass was lower in individuals from forest fragments compared with conspecifics from continuous forests (Habel *et al*., 2014). Suorsa and colleagues found that body condition was lower in Eurasian treecreeper (*Certhia familiaris*) nestlings that were raised in smaller compared to nestlings raised in larger forest patches (Suorsa *et al*., 2003, 2004). However, body condition did not change with habitat deterioration in Taita thrushes (*Turdus helleri*; Lens *et al*., 2002).

In mammals, the effect of habitat disturbance on nutritional status also varies considerably, and the limited number of studies undertaken precludes identification of a general trend. For example, food limitation, measured by the urinary urea nitrogene:creatinine ratio, increased in New England cottontails (*Sylvilagus transitionalis*) with decreasing home range patch (Villafuerte *et al*., 1997). However, in wood mice (*Apodemus sylvaticus*), body condition was not related to the size of fragments in which the animals lived (Diaz *et al*., 1999). Body masses of agile antechinus (*Antechinus agilis*) may increase or decrease in individuals from disturbed habitats, depending on sex and season (Johnstone *et al*., 2010; 2011; 2014).

As bats represent one of the most diverse and species-rich order of mammals, replicating our study in other bats species could help to better understand the impact of habitat alteration on body condition in mammals. According to a broad study on insect abundance at the study site, potential prey for insectivorous bats is reduced at the more disturbed logged sites (Ewers *et al*., 2015). Thus, it is not surprising, that in our study, habitat deterioration led to a reduced body mass in some bat species, even though bats are much more mobile than most other terrestrial mammals and thus could potentially compensate for a reduction in food availability by visiting alternative, presumably more distant, foraging areas. Indeed, our findings indicate that bat species were differently affected by habitat disturbance depending on their roost preference, which may be caused by varying home range sizes. In foliage-roosting bat species, for example, body mass was lower in actively logged and fragmented forest compared to conspecifics from lesser disturbed recovering forest.

Nutritional stress, reflected by a loss in body mass, can lead to an increased susceptibility to pathogens (Plowright *et al*., 2008). For example, in female Brazilian free-tailed bats (*Tadarida brasiliensis*) a low body mass was associated with a higher risk for rabies infection (Turmelle *et al*., 2010). Further, Lau *et al*. (2010) found an association of a coronavirus detection rate and low body mass in Chinese horseshoe bats (*Rhinolophus sinicus*). Thus, foliage-roosting bat species in disturbed habitats might become especially susceptible to viral infections and present increased shedding, potentially leading to zoonotic spillover events when they come into contact with humans or livestock.

### Chronic stress in disturbed habitats

In many vertebrates, especially mammals, N/L-ratios are positively correlated with the plasma concentration of circulating stress hormones (Davis *et al*., 2008). Chronically elevated levels of glucocorticoids shift the numbers of neutrophils and lymphocytes, causing an increase in the N/L-ratio making it a suitable measurement of chronic stress (Davis *et al*., 2008; Ellis *et al*., 2012). We used this ratio as a proxy for stress level in our study instead of directly measuring plasma glucocorticoid concentrations because hormone concentrations change within 3–5 min after capture in mammals (Romero, 2004), whereas N/L-ratios remains stable after capture and handling (Davis, 2005; Davis *et al*., 2008). Further, we were limited in the amount of available plasma given ethical considerations for the study taxa.

In one of our study species, N/L-ratios differed between individuals of different habitat types. *Rhinolophus borneensis* from fragmented forest showed higher N/L-ratios than conspecifics from actively logged forests. Similarly, in another small mammal, the agile antechinus, Johnstone and colleagues found that the N/L-ratio of individuals living in fragmented areas was elevated throughout most of the year, although not during winter (Johnstone *et al*., 2012a, 2014). In birds, results are inconsistent. On the one hand, Suorsa *et al*. (2004) and Hinam and Clair (2008) reported higher heterophil to lymphocyte ratios (H/L; the avian equivalent to N/L-ratio) in Eurasian treecreeper nestlings and Northern sawwhet owls (*Aegolius acadius*) in smaller compared to larger forest patches. On the other hand, in male ovenbirds (*Seiurus aurocapilla*) fragmentation was associated with lower H/L-ratios compared to males from the continuous forest, even though only during the breeding season (Mazerolle and Hobson, 2002).

In contrast to our hypothesis, we found an association between chronic stress and habitat disturbance in a small, highly social, cave-roosting species, but not in the larger, foliage-roosting species (*R. trifoliatus* and *R. sedulus*). Foliage-roosting species may be less resilient to forest modification and individuals suffering from chronic stress may have emigrated or deceased of starvation or disease already at the early beginning of habitat modification. In the consequence, we would not have been able to sample them.

Indeed, the total number of captured individuals was generally lower in actively logged and fragmented compared with recovering forest for most species including *R. sedulus*, which was completely absent in fragmented forest (although sampling effort was similar in all habitat types). Thus, this species may be highly vulnerable to local extinction in human-modified habitats. Due to the fact that a small home range size increases extinction risk in bats (Jones *et al*., 2003) one should attribute particular attention on species with small home ranges for conservation management.

### Effect of habitat alteration on total white blood cell counts

In three rhinolophid bat species, we used TWBC counts as a measure of the cellular immunity. White blood cells (leukocytes) are energetically costly to produce and maintain and commonly used in comparative field research (Nunn, 2002; Young *et al*., 2013; Schneeberger *et al*., 2013a; Tian *et al*., 2015). Compared to similar-sized insectivorous bat species from the Neotropics, we found low mean values of leukocytes per field. Schneeberger *et al*. (2013a) reported between 1.8 (*Micronycteris microtis*) and 3.9 (*M. hirsuta*) leukocytes per visual field while the range in our study was only 0.27–0.32 leukocytes per field. Stockmaier *et al*. (2015) reported similar numbers of leukocytes (0.85) in the similar-sized, insectivorous Pallas’ mastiff bat (*Molossus molossus*).

In one species, *R. sedulus*, we found a significant effect of habitat disturbance, with significantly lower numbers of leukocytes in individuals from actively logged than individuals from recovering forests, which is consistent with findings in diademed sifaks (*Propithecus diadema*; Irwin *et al*., 2010) and agile antechinus (Johnstone *et al*., 2012b). In these other studies, authors suggested that animals suffered from an impaired immune system function, yet no experimental tests were performed on functional aspects of the immune system in support of this notion. High numbers of leukocytes can either indicate an individual in good condition that is well prepared to fight an infection or an individual currently fighting an infection (Salvante, 2006). Because all animals appeared healthy on visual examination, we support the former explanation and suggest that individuals in actively logged forest suffered from a weakened immune system. In support with this, a previous study on Brazilian free-tailed bats showed that individuals roosting in certain disturbed locations (human-made bridges) can have lower cellular immune function compared to individuals roosting in natural caves (Allen *et al*., 2009).

In *R. sedulus* a large proportion of leukocytes consisted of lymphocytes (36.73 ± 24.28%), a cell type that is used to counter viral infections by killing infected cells and the activation of the cytokine and antibody response (Baker and Zhou, 2015). Therefore, *R. sedulus* with low leukocyte numbers might be particularly prone to shed pathogens, potentially causing zoonotic spillovers when humans come into contact with this species. In Southeast Asia, besides flying foxes also larger insectivorous bats are occasionally hunted for their meat (Mildenstein *et al*., 2016), as it may be the case for *R. sedulus*.

*Rhinolopohus sedulus* seems to be less resilient to habitat disturbance compared with *R. borneensis* and *R. trifoliatus* that might develop reduced numbers of leukocytes during later stages of habitat conversion. Like *R. trifoliatus*, but contrasting to *R. borneensis*, *R. sedulus* is smaller compared to the other congeneric species. Although N/L-ratios of individuals in *R. sedulus* were not elevated in actively logged compared to recovering forest, a non-stress related mechanism affecting all immune cells in a similar way might have led to the reduction in TWBC counts.

## Conclusions

Overall, we found evidence that in some, but not all studied paleotropical bat species human habitat disturbances may be associated with a decrease in body mass, elevated chronic stress and reduced investment in cellular components of the immune system. We identified foliage-roosting species as particularly sensitive to habitat deterioration, which may predispose them as potential sources for viral spillovers events when humans encroach into natural habitats. Presumably, a prolonged period of habitat disturbances will result in chronic stress and an impaired immune system in more species, especially because forest modification during our study only lasted for a short period of time. A follow-up study at a later stage of fragmentation may clarify whether and which bat species are resilient to human disturbances over a longer period.
